# Effect of Class II functional treatment on facial attractiveness, as perceived by professionals and laypeople

**DOI:** 10.1038/s41598-021-93343-0

**Published:** 2021-07-07

**Authors:** Federica Santori, Francesco Masedu, Domenico Ciavarella, Edoardo Staderini, Claudio Chimenti, Michele Tepedino

**Affiliations:** 1 Department of Biotechnological and Applied Clinical Sciences, University of L’Aquila, V.Le San Salvatore, Edificio Delta 6, 67100 L’Aquila, Italy; 2 Department of Clinical and Experimental Medicine, University of Foggia, Foggia, Italy; 3 Fondazione Policlinico Universitario A. Gemelli IRCCS, Roma-Università Cattolica del Sacro Cuore, Istituto di Clinica Odontoiatrica e Chirurgia Maxillo-Facciale, Rome, Italy

**Keywords:** Dentistry, Orthodontics

## Abstract

The literature offers different perspectives for and against two-phase treatment of skeletal Class II malocclusion. Facial attractiveness is an important aspect to take into account, given that children with skeletal Class II are often bullied by their peers and have low self-esteem and a lower social perception. The aim of the present study was to evaluate the aesthetic perception of facial profiles by a large number of observers, before and after treatment with a functional appliance, compared to untreated controls. The pre- and post-treatment cephalograms of 20 Class II subjects treated with Sander’s bite-jumping appliance and 20 untreated historical controls were collected and transformed into black and white silhouettes depicting only the lower third of the face. An online questionnaire comprising the silhouettes of the two groups, three “calibration” profiles and an “ideal” profile was submitted to dentists, orthodontists, undergraduates and laypeople, asking them to rate the profile’s attractiveness using a Visual Analogue Scale (VAS). The effect of treatment, and observers’ age, expertise and gender were analysed. The calibration images and the ideal profiles were used to evaluate the coherence of each observer’s judgement. The protocol was approved by the local Ethics Committee. Nine-hundred and ten questionnaires were collected. Treated subjects showed a larger improvement of facial attractiveness compared to controls. A significant effect of gender on the observer’s ratings was observed. Some observers showed incoherent judgement, which had a significant effect on the regression model. In conclusion, early treatment with functional appliances seems to improve patients’ facial aesthetics. This improvement is perceived equally by dental professionals and laypeople.

## Introduction

Class II skeletal malocclusion is a relatively common condition, characterized by a skeletal disharmony that usually results in a reduced chin projection and a convex facial profile^1^. These characteristics, in addition to other typical features of Class II malocclusions, like protruded upper incisors, a decreased mentolabial angle, a retruded lower lip, and a short chin–throat distance, could impair the subject’s facial attractiveness^1–3^.

Several studies have reported that an unbalanced facial appearance could negatively affect both self-esteem and social perception, with an impact on bullying and career opportunities^4–6^. In fact, patients with an attractive smile are perceived as more intelligent and easily employed compared to subjects with an unattractive dentition^7,8^, have higher chances of getting a better job position, and are perceived as having a better socioeconomic status^6^.

Consequently, facial attractiveness should be one of the treatment goals for Class II skeletal malocclusion, along with correct dental occlusion and a functional equilibrium. However, according to the current literature such aesthetic balance is not always achieved. While some authors reported facial improvement in young adults after treatment with the Herbst appliance^9^ and in children after functional treatment with a Twin-Block device^10^, others observed no perceived difference in facial attractiveness after treatment^11^.

Such results are not surprising, given that the literature is not even in agreement about the skeletal outcome of Class II functional appliance treatment. While some authors have reported a significant increase in mandibular length after treatment with functional appliances compared to controls^12^, another systematic review by Koretsi et al. indicated that the observed outcomes were mostly dentoalveolar rather than skeletal^13^. However, it should be noted that the latter review included many studies involving patients in a pre-pubertal skeletal growth stage, which is known to affect treatment efficacy^14^. A Cochrane systematic review concluded that, when comparing the outcome of one-phase treatment (the use of a fixed appliance in a post-pubertal stage) to those of two-phase treatments (the use of functional appliances during the pubertal stage, followed by treatment with fixed appliances), the only advantage that could favour early treatment with a functional appliance is a reduction in the incidence of upper incisors trauma^15^.

Based on these considerations, two questions arise: is early treatment with a functional appliance able to improve the aesthetic profile of the patient? Is this improvement perceived by the patient and does it improve their social interactions? Different studies have tried to answer the first question, with contradictory results^16–18^. Similarly, many authors have tried to evaluate how the aesthetic profile is perceived by professionals and laypeople^6,19–24^, reaching different conclusions.

The aim of the present study was to involve a large number of observers with different expertise—laypeople, dental students, general dentists and orthodontists—to evaluate the aesthetic perception of pre- and post-treatment facial profiles of patients with skeletal Class II treated with a removable functional appliance, compared to untreated controls.

## Materials and methods

The present protocol was designed following the recommendations of the Declaration of Helsinki from 1975 and subsequent revisions, and was approved by the Internal Review Board of the University of L’Aquila (protocol no 15352, approval no 02/21). All the participants gave their written informed consent to participate.

### Selection of the sample of skeletal Class II subjects

The clinical records of patients treated at the Dental Clinic, University of L’Aquila, Italy, from January 2005 to March 2020, were screened in chronological order for the following inclusion criteria: skeletal Class II malocclusion with ANB > 4° (the angle between maxillary A point, the skeletal Nasion point, and the mandibular B point), Class II division 1 with full-cusp molar Class II dental relationship orthodontic treatment with a Sander bite-jumping appliance, treatment onset at a CS3 cervical vertebral maturation (CVM)^25^ stage, high-quality pre- and post-treatment radiographic documentation, with clearly distinguishable soft tissues, and a treatment outcome that was considered successful by achieving a molar Class I. The screening ceased when the first 20 cases were selected. The demographic data (age and gender) and the pre-treatment (T0) and post-treatment (T1) lateral cephalograms were extracted from the records, anonymized and stored for further analysis. An inactive control group, of the same size (n = 20) and matched for ANB value, age, gender distribution, and CVM stage, was selected from the records of the American Association of Orthodontists Foundation (AAOF) craniofacial growth legacy collection (http://www.aaoflegacycollection.org/). For the control group, the T0 cephalogram was taken at an age comparable to that of the T0 of the study group, while the T1 cephalogram was selected after a time interval corresponding to the mean treatment time of the study group (24 months).

**Figure 1:**
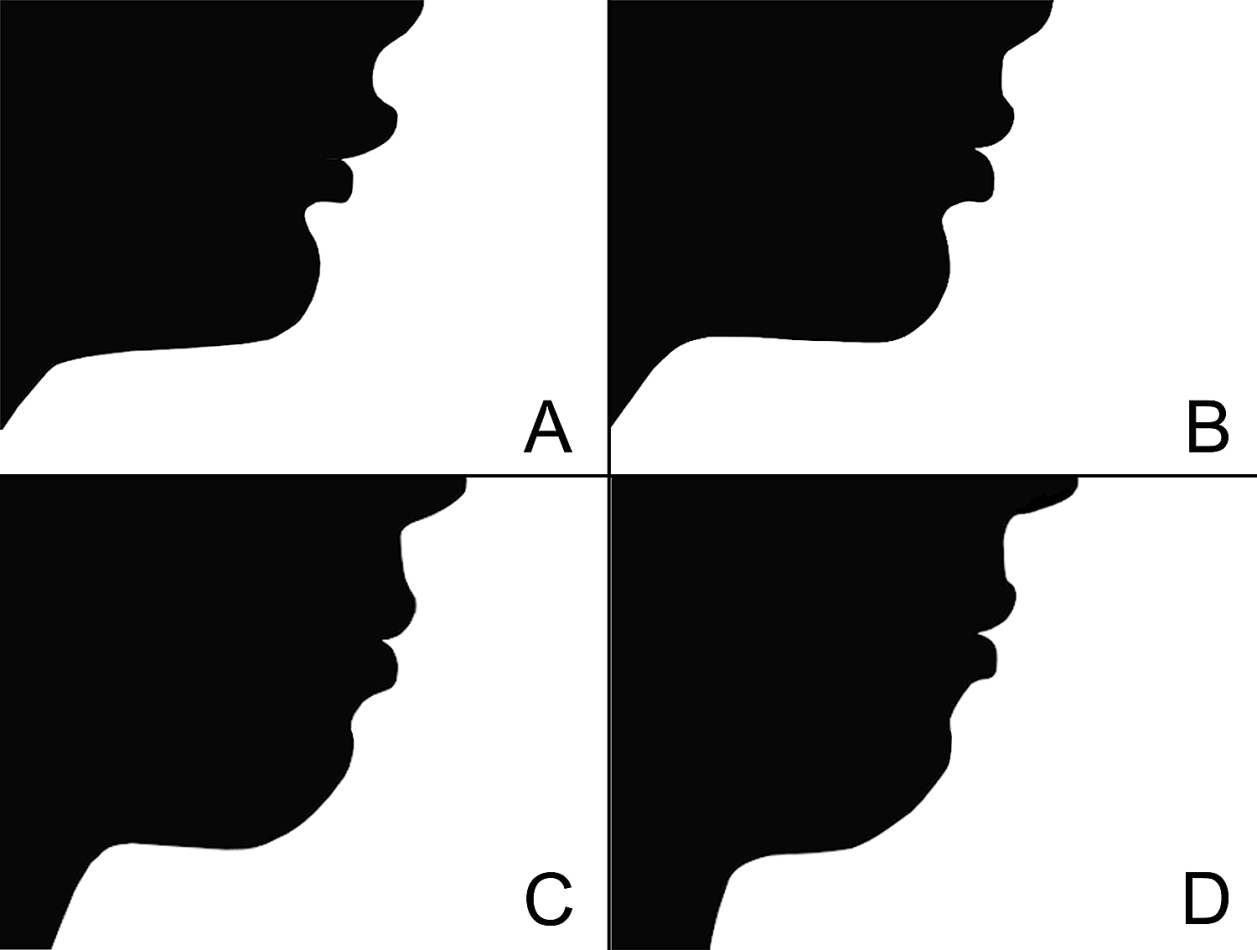
Examples of silhouettes retrieved from patients’ profiles and included in the questionnaire: T0 (**A**) and T1 (**B**) silhouettes of a patient treated with Sander’s bite jumping appliance, and T0 (**C**) and T1 (**D**) silhouettes of an untreated control patient.

All the cephalograms were oriented with the Frankfurt plane parallel to the ground and then transformed into black and white silhouettes depicting the soft tissue facial profile, using image editing software (GNU Image Manipulation Program version 2.10, Free Software Foundation Inc., Boston, MA, USA). Each image was then cropped superiorly 1 cm above the subnasale point, anteriorly 2 cm away from the tip of the nose, inferiorly 4 cm below the throat point, and posteriorly through the tragus point (Fig. 1).

### Preparation of the online questionnaire

**Figure 2:**
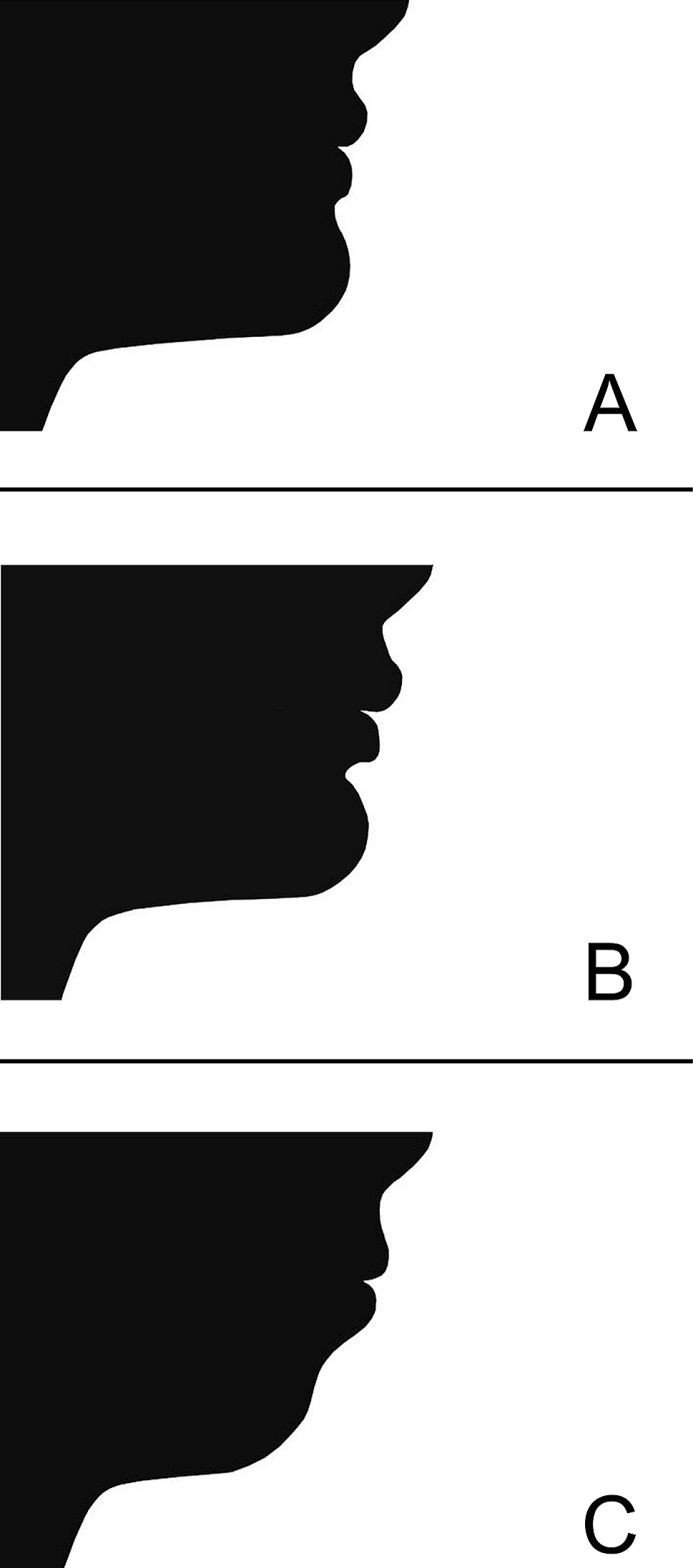
Calibration silhouettes showed to the observers for evaluation. (**A**) Calibration profile no. 1 depicting a “very pleasant” profile; (**B**) calibration profile no. 2 depicting a “medium” profile; (**C**) calibration profile no. 3 depicting a “very unpleasant” profile.

**Figure 3:**
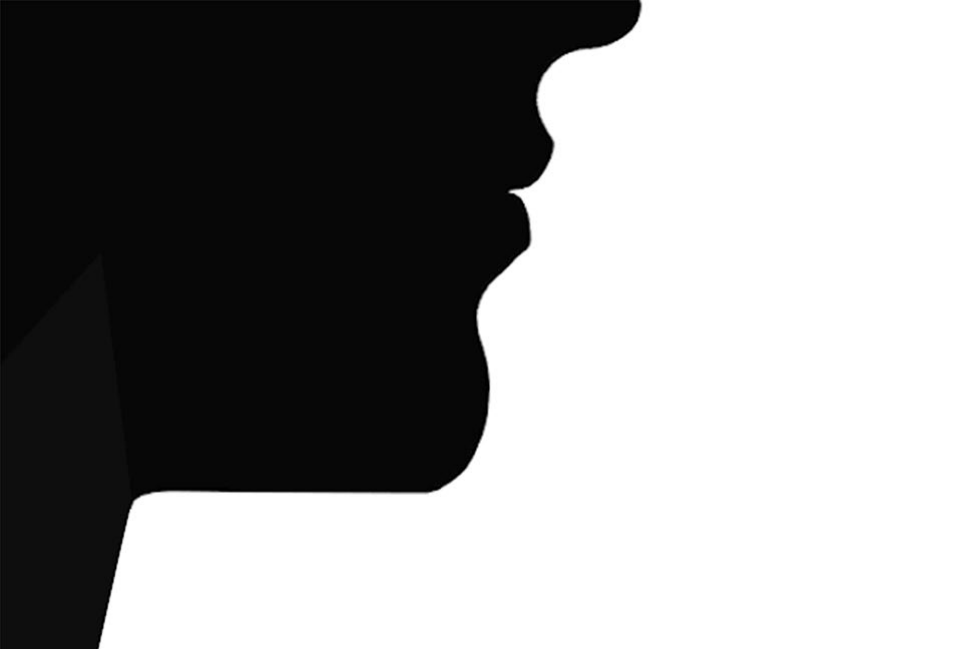
Silhouette of the “ideal” profile redrawn from the publication of Czarnecki et al., 1993.

A four-page online questionnaire was then prepared using an online service (http://www.surveymonkey.com/): the first page of the questionnaire was used to inform the responder about the study and obtain their informed consent; no information was given about the nature of the images and the scope of the study, to ensure blinding of the observers. The second page was used to collect information about gender, age, profession, and years of experience in the case of dental professionals. The third page presented three “calibration” images, with the intention to show three attractiveness categories, from the least attractive to the most pleasant. The three calibration images were obtained from the same radiograph of a subject with a mild skeletal Class II (ANB = 4.6°), a marked labio-mental angle, but a pleasant lip projection; this image was used as the “medium” reference (Fig. 2B). Then, the same image was modified by retruding the chin markedly to obtain a “very unpleasant” profile (Fig. 2C), and by moving the chin forward to simulate a skeletal Class I and modifying the lips to conform to the cephalometric norms^23^ to obtain a “very pleasant” profile (Fig. 2A). The fourth page showed the T0 and T1 pictures of the study group and the control group in a random order (a random sequence of numbers was obtained using an online tool www.randomizer.org) and without any identifier, plus one “ideal” profile (Fig. 3) showing the perfect proportions suggested by cephalometric norms and redrawn from a previous publication^23^. The observers were asked to rate the attractiveness of the image shown by using a visual analogue scale (VAS) bar from 0 (absolutely not attractive) to 10 (absolutely attractive). The meaning of the VAS score was explained only in the first page of the questionnaire.

The questionnaire was disseminated online through social media to Italian people of any age and expertise, for 2 months. Taking the VAS score associated with the profiles by the observers as the primary endpoint and setting the null hypothesis H_0_: δ = 0, we calculated a-priori that 745 observers would be adequate assuming 90% power, 1% first type error, and a 0.2 Cohen threshold. After adjusting this number for an expected 10% rate of incomplete questionnaires, an overall sample size of 820 observers was defined.

### Observers’ coherence

To evaluate each observer’s ability to perform a coherent aesthetic assessment, and to gain an impression of the overall quality of the scores, two binary variables were created: the external coherence, that is the ability to discriminate the ordinal categories of the three calibration profiles, and the internal coherence, that is the consistency in assigning the “very pleasant” calibration profile and the “ideal” profile to the same ordinal category of scores (i.e. internal coherence is present if the score assigned to the “ideal” profile is greater than the score given to the “medium” calibration profile by the same observer). An observer was considered incoherent if their external coherence and internal coherence had different values (i.e. externally coherent but internally incoherent, or vice-versa).

### Statistical analysis

Descriptive statistics were calculated for all the variables. A Shapiro–Wilk normality test was used to assess the type of data distribution for all the variables. The ANB value and the age of the subjects at T0 in the study group and in the control group were compared using a T-test for independent samples. The gender distribution between the two groups was compared using a Pearson χ^2^ test. After a test for the equality of variances, a T-test for independent samples was performed to compare the T1–T0 differences in scores between the two groups. To evaluate the effects of treatment, observer’s age, gender, expertise, and coherence on the VAS scores, a multivariate random intercept model was performed.

To further analyse observers’ coherence, a Shapiro–Wilk test was used to compare the skewness of the scores attributed to the “ideal” profile by the coherent and the incoherent observers. The distribution of incoherent observers among genders and expertise was also studied through a Pearson χ^2^ test. Then, to gain an impression of the quality of the incoherent scores (that is, are they an expression of the observer’s very own perception or are they due to a random assignment of scores), a one-sample T-test for proportion was used to test the null hypothesis that the distribution of the two types of coherence among the incoherent observers was different from 0.5.

## Results

The demographic characteristics of the skeletal Class II patients included in the treated group and in the control group are reported in Table 1. The two groups were comparable for gender distribution (Pearson χ^2^ = 0.032, p = 0.858), age at T0 (independent samples T-test, mean difference − 0.14, p = 0.757, assuming unequal variances) and age at T1 (independent samples T-test, mean difference 0.01, p = 0.990, assuming unequal variances). The Treatment group showed a mean ANB value of 5.9° ± 1.4, the Control group showed a mean ANB value of 5.6° ± 1.7, all being normally distributed, with no statistical difference between the two groups (independent samples T-test assuming equal variances, mean difference − 0.3°, p = 0.559).

**Table 1:** Demographic characteristics of the skeletal class II treated and untreated groups.

Timepoint	Treated group		Control group	
	Male (n = 10)	Female (n = 10)	Male (n = 9)	Female (n = 11)
T0	12.5 ± 1.7 (0.415)	10.9 ± 1.6 (0.534)	11.9 ± 0.5 (0.564)	11.2 ± 0.8 0.434)
T1	14.5 ± 2.3 (0.443)	12.9 ± 2.1 (0.252)	14.3 ± 0.9 (0.115)	13.1 ± 0.9 (0.780)

Mean ± standard deviation (p value from Shapiro–Wilk normality test). Age expressed in years.

A total of 910 questionnaires were collected, with a completion rate of 61%. Thirty-eight percent of the responders (n = 343) were male and 62% (n = 567) were female. The majority of the responders (36%, n = 328) were aged between 25 and 34 years old; 18% (n = 167) were aged between 35 and 44 years old, 17% (n = 159) between 18 and 24 years old, 13% (n = 118) between 55 and 64 years old, 12% (n = 109) between 45 and 44 years old, and 3% (n = 29) above 65 years old. Regarding the responders’ expertise, half of the observers (n = 452) were laypeople, 24% (n = 214) were dentists, 18% (n = 168) were orthodontists, and 8% (n = 76) were undergraduate students in dentistry.

The descriptive statistics for the VAS scores attributed by the observers to the three calibration profiles, to the ideal profile, and to the Treatment group and the Control group profiles are reported in Table 2.

**Table 2:** Descriptive statistics for the overall VAS scores, divided by the observer's expertise.

Subject	Observer	Gender	Shapiro–Wilk normality test	Mean	Standard deviation	Minimum score	Maximum score
Ideal profile	Dentist (n = 214)	Male (n = 101)	0.136	5.55	1.8	1.00	10.00
		Female (n = 113)	0.003	5.33^a^	2.4	0.00	10.00
	Orthodontist (n = 168)	Male (n = 45)	0.083	6.00	1.2	3.00	9.00
		Female (n = 123)	< 0.001	5.56^a^	2.2	0.00	10.00
	Undergraduate (n = 76)	Male (n = 34)	0.176	4.67	2.6	0.00	9.00
		Female (n = 42)	0.086	5.36	2.5	0.00	10.00
	Laypeople (n = 452)	Male (n = 163)	< 0.001	5.09^a^	2.2	0.00	10.00
		Female (n = 289)	< 0.001	4.84^a^	2.4	0.00	10.00
Calibration profile 1 "very pleasant"	Dentist (n = 214)	Male (n = 101)	0.001	6.37^a^	2.2	2.00	10.00
		Female (n = 113)	0.004	6.03^a^	2.1	0.00	10.00
	Orthodontist (n = 168)	Male (n = 45)	0.072	6.78	1.9	3.00	10.00
		Female (n = 123)	< 0.001	6.31^a^	2.2	0.00	10.00
	Undergraduate (n = 76)	Male (n = 34)	0.001	6.64^a^	2.2	0.00	9.00
		Female (n = 42)	0.079	6.17	2.0	2.00	10.00
	Laypeople (n = 452)	Male (n = 163)	< 0.001	6.16^a^	2.1	0.00	10.00
		Female (n = 289)	< 0.001	6.21^a^	2.2	0.00	10.00
Calibration profile 2 "medium"	Dentist (n = 214)	Male (n = 101)	0.010	4.58^a^	2.4	0.00	10.00
		Female (n = 113)	0.005	3.84^a^	2.2	0.00	9.00
	Orthodontist (n = 168)	Male (n = 45)	0.070	3.73	2.2	0.00	10.00
		Female (n = 123)	0.001	3.45^a^	2.0	0.00	8.00
	Undergraduate (n = 76)	Male (n = 34)	0.070	4.73	2.1	0.00	8.00
		Female (n = 42)	0.163	4.60	2.1	0.00	8.00
	Laypeople (n = 452)	Male (n = 163)	0.002	4.37^a^	2.3	0.00	10.00
		Female (n = 289)	< 0.001	4.44^a^	2.3	0.00	10.00
Calibration profile 3 "very unpleasant"	Dentist (n = 214)	Male (n = 101)	< 0.001	1.72^a^	1.6	0.00	7.00
		Female (n = 113)	< 0.001	1.11^a^	1.3	0.00	6.00
	Orthodontist (n = 168)	Male (n = 45)	< 0.001	1.15^a^	1.0	0.00	4.00
		Female (n = 123)	< 0.001	0.94^a^	1.1	0.00	5.00
	Undergraduate (n = 76)	Male (n = 34)	0.001	1.88^a^	1.5	0.00	7.00
		Female (n = 42)	< 0.001	1.19^a^	1.2	0.00	5.00
	Laypeople (n = 452)	Male (n = 163)	< 0.001	2.04^a^	1.8	0.00	9.00
		Female (n = 289)	< 0.001	1.71^a^	1.7	0.00	9.00
Treatment group T0	Dentist (n = 214)	Male (n = 101)	0.215	3.66	1.1	1.40	6.40
		Female (n = 113)	0.653	2.90	1.4	0.25	6.00
	Orthodontist (n = 168)	Male (n = 45)	0.817	3.53	0.9	1.85	5.35
		Female (n = 123)	0.076	3.09	1.3	0.10	5.50
	Undergraduate (n = 76)	Male (n = 34)	0.173	3.56	1.6	1.05	7.69
		Female (n = 42)	0.066	2.68	1.0	0.70	6.15
	Laypeople (n = 452)	Male (n = 163)	0.065	3.43	1.3	1.05	7.90
		Female (n = 289)	0.005	2.90^a^	1.4	0.00	6.38
Treatment group T1	Dentist (n = 214)	Male (n = 101)	0.638	4.46	1.2	1.75	6.90
		Female (n = 113)	0.418	4.04	1.3	1.30	6.80
	Orthodontist (n = 168)	Male (n = 45)	0.875	4.72	0.9	2.70	6.25
		Female (n = 123)	0.001	4.33^a^	1.4	0.60	6.50
	Undergraduate (n = 76)	Male (n = 34)	0.810	4.06	1.6	1.30	7.71
		Female (n = 42)	0.758	3.84	1.2	0.59	6.50
	Laypeople (n = 452)	Male (n = 163)	0.319	4.20	1.3	1.65	7.90
		Female (n = 289)	0.003	3.84^a^	1.5	0.15	7.50
Control group T0	Dentist (n = 214)	Male (n = 101)	0.330	4.04	1.0	2.00	6.53
		Female (n = 113)	0.084	3.50	1.2	1.06	6.53
	Orthodontist (n = 168)	Male (n = 45)	0.938	4.28	0.8	2.53	5.82
		Female (n = 123)	0.005	3.92^a^	1.4	0.71	6.59
	Undergraduate (n = 76)	Male (n = 34)	0.157	3.72	1.5	1.57	7.75
		Female (n = 42)	0.985	3.34	1.2	0.42	6.12
	Laypeople (n = 452)	Male (n = 163)	0.438	3.68	1.3	1.06	7.88
		Female (n = 289)	0.001	3.28^a^	1.5	0.00	6.59
Control group T1	Dentist (n = 214)	Male (n = 101)	0.739	4.07	1.1	1.82	6.65
		Female (n = 113)	0.381	3.52	1.2	0.41	6.76
	Orthodontist (n = 168)	Male (n = 45)	0.090	4.21	0.9	2.18	5.40
		Female (n = 123)	0.004	3.87^a^	1.4	0.24	6.47
	Undergraduate (n = 76)	Male (n = 34)	0.302	3.70	1.5	0.94	7.93
		Female (n = 42)	0.296	3.43	1.1	1.14	6.59
	Laypeople (n = 452)	Male (n = 163)	0.227	3.75	1.3	1.18	7.94
		Female (n = 289)	0.027	3.46^a^	1.4	0.29	7.18

^a^Non-normal distribution according to the Shapiro–Wilk normality test. To improve the readablity of the results, for each observer a mean value of all the scores submitted for each group at T0 and T1 was used to calculate the present descriptive statistics.

The independent samples T-test adjusted for the lack of homoscedasticity revealed a statistically significant difference between the difference in T1–T0 scores assigned by all the observers overall to the Treatment group compared to the Control group (Table 3). The random intercept model revealed a significant interaction of the presence/absence of treatment, the gender of the observers, and the observers’ coherence on the VAS scores attributed to the profiles (Table 4).

**Table 3:** Statistical comparison for the T1–T0 difference in VAS scores from all the observers between the Treatment group and the Control group.

Group	Test for homoscedasticity	Observations	Mean	Standard deviation	Mean difference^†^	P value^†^	95% Confidence interval^†^	
							Lower bound	Upper bound
Control	0.011	15,470	0.16	2.2	− 0.47**	< 0.001	− 0.52	− 0.42
Treatment		18,200	0.63	2.2				

**Statistically significant with p < 0.01; ^†^Values from independent samples T-test with Satterthwaite's correction for the lack of homoscedasticity.

**Table 4:** Random intercept model.

Dependent variables	Coefficient	Standard error	z	P value	95% Confidence interval	
					Lower bound	Upper bound
Treatment	0.469	0.02	19.53**	< 0.001	0.42	0.52
Age	− 0.011	0.01	− 1.11	0.268	− 0.03	0.01
Gender	0.119	0.03	4.33**	< 0.001	0.06	0.017
Expertise	− 0.019	0.01	− 1.65	0.098	− 0.04	0.01
Coherence	0.328	0.03	12.16**	< 0.001	0.27	0.38
Constant	0.031	0.06	0.55	0.585	− 0.08	0.14

*Statistically significant with p < 0.05; **statistically significant with p < 0.01; Model fitting: Wald χ^2^ = 560.3, p < 0.001.

Regarding the coherence analysis, a total of 27.4% of the observers were incoherent. The distribution of incoherent observers was significant among gender, but not among different types of expertise (Supplementary File 1). Interestingly, the scores attributed to the “ideal” profile by coherent and incoherent observers showed a contrasting trend (Supplementary File 2). The one sample test of proportion (Supplementary File 3) revealed a distribution significantly different from 0.5 (mean 0.92, z = 13.4, p < 0.001).

## Discussion

To the best of our knowledge, this is the first study of this kind to collect a large sample of observers and have them evaluate the attractiveness of a meaningful sample of treated and untreated profiles. The Treatment and the Control group were comparable in terms of age, gender distribution, and skeletal Class II severity evaluated through the ANB angle, thus reducing the number of possible confounders. Treatment with a Sander bite-jumping appliance was chosen because, according to a systematic review, it is the removable appliance that seems to produce the greatest increase in Condilion-Pogonion mandibular length^12^.

The use of black and white silhouettes has been suggested by several authors, because they overcome the effect of confounding factors like hair, eyes, skin color, makeup etc.^19,26^, and are especially suitable to evaluate the sagittal position of the jaws^11^. In addition, in the present study the silhouettes were cropped at the level of the subnasale point, to allow the observers to focus on the lower third of the face, without being distracted by the shape and dimension of the nose^27^ or the forehead.

The use of VAS scores is a simple method that is easily understood and applied by the observers and widely approved by the scientific community for tasks like the one pursued in the present study^11,26^.

In general, the mean scores attributed by the observers were in the middle of the VAS range. This could have been expected, since it is known that skeletal Class II profiles are considered less attractive than Class I profiles^2,6^. On the other hand, it was surprising that the “very pleasant” profile among the three calibration images and the “ideal” profile also scored slightly above the average (Table 2). Indeed, only 54 observers attributed a score of 10 to the “very pleasant” calibration profile, and only 13 observers out of 612 assigned a score of 10 to the “ideal” profile. Furthermore, there was a difference in scoring the “ideal” profile between the coherent and incoherent observers (Supplementary File 2). Indeed, the observers’ coherence had a significant effect on the VAS scores collected (Table 3), and according to the statistical analysis it is likely that the incoherence, as defined in the present work, was an expression of the inner scheme of categorization of each observer, rather than an expression of an inaccuracy in scoring. However, it must be noted that the meaning of the variable coherence can be subjected to different psychometric interpretations, which are beyond the scope of the present manuscript.

Overall, the Treatment group showed a greater T1–T0 increase in VAS scores than the Control group, based on all observers (Table 3). It is worth remarking that despite this effect seems small, it corresponds to the 9% of the average score of the “ideal” profile observed in the present study. This result suggests that functional treatment is effective at improving a patient’s facial attractiveness, compared to the absence of treatment. This is consistent with previous studies conducted on smaller samples^9,10,19,24,28^ and in contrast with another study that reported no differences between the pre-treatment and the post-treatment profiles^11^. There were no differences in facial attractiveness perception between dentists, orthodontists, undergraduates in dentistry, and laypeople. This finding is in contrast with previous studies^3,9,21,22,29,30^. On the other hand, other authors reported results similar to those of the present study^6,11,19,24,31–33^. Similarly, the observer’s age had no significant effect on the ratings assigned to the profiles. Interestingly, a strong effect of gender on the aesthetic perception of the observers was detected, with male observers tending to assign higher scores than females. This finding is in contrast with that of O’Neill et al.^11^ who found no differences between male and female raters.

Even though it is possible to conclude with adequate confidence that Class II treatment with functional appliances improves the aesthetic perception of the profile by both laypeople and professionals, although with gender differences, an evaluation of the impact on patient’s quality of life would be advisable so that this aspect can be taken into account when recommending two-phase treatment.

### Limitations

The rate of complete responses was lower than expected, but this was understandable since the observers were asked to evaluate a very large number of profiles (n = 84). Missingness was assumed missing completely at random (MCAR) based on the distribution shapes of random sub-samples bootstrapped from the overall sample.

Disseminating a questionnaire online provided several advantages^34^, but on the other hand the responses were not collected in a standardized manner and it was impossible to control such factors like the time spent on each image, or whether the observers completed the questionnaires with the help of their peers. In addition, it was not possible to change the random order of the silhouettes for every observer due to technical limitations. Under this point of view, the coherence test provided assumed a crucial role. Dissemination of the questionnaire was limited to people living in Italy, because cultural differences could act as confounders^26,35,36^; although this strengthens the validity of our results, it reduces their applicability to other populations with a different cultural background.

The use of historical controls has been criticized by some authors because growth is influenced by secular trends^37^. However, it is the only ethically acceptable method and other authors have concluded that their use offers results comparable to those of a prospectively recruited sample^38^.

## Conclusions

Skeletal Class II treatment with a Sander’s bite-jumping appliance is effective at improving the patient’s profile attractiveness, compared to untreated Class II controls. This improvement is perceived equally by dental professional with different levels of expertise and laypeople. On the other hand, a difference between gender was observed, with female observers assigning lower scores. Many observers showed incoherence when evaluating the three calibration profiles and the ideal profile; this finding was gender-related and had a significant impact on the results, but was considered an expression of grading ability rather than an inaccuracy in scoring.

## Supplementary Information

Supplementary Legends.

Supplementary Information 1.

Supplementary Information 2.

Supplementary Information 3.

## Data Availability

The datasets used and/or analysed during the current study are available from the corresponding author on reasonable request.
