# Supplementary material for: Effect of Class II functional treatment on facial attractiveness, as perceived by professionals and laypeople
Source: Sci Rep. 2021 Jul 7;11:13989. doi: 10.1038/s41598-021-93343-0 (PMC8263773; doi:10.1038/s41598-021-93343-0)
Supplement: Supplementary file 1 — Supplementary Legends. [file 41598_2021_93343_MOESM1_ESM.docx]

**CAPTIONS TO SUPPLEMENTARY FILES 2 AND 3**

**Supplementary file 2.** A Shapiro-Wilk test was used to compare the skewness of score distribution between the coherent and incoherent subjects. The two groups showed an opposite trend in score assignment to the “ideal” profile. **A**, histogram showing the distribution of scores assigned from “incoherent” subjects to the “ideal” profile; **B**, histogram showing the distribution of scores assigned from “coherent” subjects to the “ideal” profile

**Supplementary file 3.** Frequency table and one sample test of proportion for the distribution of external and internal coherence among the incoherent subjects.
