# Supplementary material for: Effect of Class II functional treatment on facial attractiveness, as perceived by professionals and laypeople
Source: Sci Rep. 2021 Jul 7;11:13989. doi: 10.1038/s41598-021-93343-0 (PMC8263773; doi:10.1038/s41598-021-93343-0)
Supplement: Supplementary file 2 — Supplementary Information 1. [file 41598_2021_93343_MOESM2_ESM.docx]

| **Table S1. Distribution of different combinations of incoherence among male subjects.** | | | | | |
| --- | --- | --- | --- | --- | --- |
|  | | | *Internal coherence* | | |
|  |  |  | *0* | *1* | *Total* |
| *External coherence* | *0* | Frequency | 12 | 8 | 20 |
|  |  | Row percentage | 60 | 40 | 100.00 |
|  |  | Column percentage | 12.77 | 6.61 | 9.30 |
|  | *1* | Frequency | 82 | 113 | 195 |
|  |  | Row percentage | 42.05 | 57.59 | 100.00 |
|  |  | Column percentage | 87.23 | 93.39 | 90.70 |
|  | *Total* | Frequency | 94 | 121 | 215 |
|  |  | Row percentage | 43.72 | 56.28 | 100.00 |
|  |  | Column percentage | 100.00 | 100.00 | 100.00 |
| *Pearson* χ*^2^ = 2.375, p= 0.123; Cramer's V= 0.105.* | | | | | |

**Supplementary file 1.** Tables describing the distribution of incoherent subjects among gender and expertise categories.

| **Table S2. Distribution of different combinations of incoherence among female subjects.** | | | | | |
| --- | --- | --- | --- | --- | --- |
|  | | | *Internal coherence* | | |
|  |  |  | *0* | *1* | *Total* |
| *External coherence* | *0* | Frequency | 22 | 11 | 33 |
|  |  | Row percentage | 66.67 | 33.33 | 100.00 |
|  |  | Column percentage | 12.94 | 4.85 | 8.31 |
|  | *1* | Frequency | 148 | 216 | 364 |
|  |  | Row percentage | 40.66 | 59.34 | 100.00 |
|  |  | Column percentage | 87.06 | 95.15 | 91.69 |
|  | *Total* | Frequency | 170 | 227 | 397 |
|  |  | Row percentage | 42.82 | 57.18 | 100.00 |
|  |  | Column percentage | 100.00 | 100.00 | 100.00 |
| *Pearson* χ*^2^ = 8.358, p= 0.004; Cramer's V= 0.145.* | | | | | |

| **Table S3. Distribution of different combinations of incoherence among dental professionals.** | | | | | |
| --- | --- | --- | --- | --- | --- |
|  | | | *Internal coherence* | | |
|  |  |  | *0* | *1* | *Total* |
| *External coherence* | *0* | Frequency | 5 | 3 | 8 |
|  |  | Row percentage | 62.50 | 37.50 | 100.00 |
|  |  | Column percentage | 11.11 | 3.23 | 5.80 |
|  | *1* | Frequency | 40 | 90 | 130 |
|  |  | Row percentage | 30.77 | 69.23 | 100.00 |
|  |  | Column percentage | 88.89 | 96.77 | 94.20 |
|  | *Total* | Frequency | 45 | 93 | 138 |
|  |  | Row percentage | 32.61 | 67.39 | 100.00 |
|  |  | Column percentage | 100.00 | 100.00 | 100.00 |
| *Pearson* χ*^2^ = 3.453, p= 0.063; Cramer's V= 0.158.* | | | | | |

| **Table S4. Distribution of different combinations of incoherence among orthodontists.** | | | | | |
| --- | --- | --- | --- | --- | --- |
|  | | | *Internal coherence* | | |
|  |  |  | *0* | *1* | *Total* |
| *External coherence* | *0* | Frequency | 1 | 2 | 3 |
|  |  | Row percentage | 33.33 | 66.67 | 100.00 |
|  |  | Column percentage | 3.33 | 2.30 | 2.56 |
|  | *1* | Frequency | 29 | 85 | 114 |
|  |  | Row percentage | 25.44 | 74.56 | 100.00 |
|  |  | Column percentage | 96.67 | 97.70 | 97.44 |
|  | *Total* | Frequency | 30 | 87 | 117 |
|  |  | Row percentage | 25.64 | 74.36 | 100.00 |
|  |  | Column percentage | 100.00 | 100.00 | 100.00 |
| *Pearson* χ*^2^ = 0.096, p= 0.757; Cramer's V= 0.029.* | | | | | |

| **Table S5. Distribution of different combinations of incoherence among undergraduate students.** | | | | | |
| --- | --- | --- | --- | --- | --- |
|  | | | *Internal coherence* | | |
|  |  |  | *0* | *1* | *Total* |
| *External coherence* | *0* | Frequency | 5 | 2 | 7 |
|  |  | Row percentage | 71.43 | 28.57 | 100.00 |
|  |  | Column percentage | 16.13 | 8.70 | 12.96 |
|  | *1* | Frequency | 26 | 21 | 47 |
|  |  | Row percentage | 55.32 | 44.68 | 100.00 |
|  |  | Column percentage | 83.87 | 91.30 | 87.04 |
|  | *Total* | Frequency | 31 | 23 | 54 |
|  |  | Row percentage | 57.41 | 42.59 | 100.00 |
|  |  | Column percentage | 100.00 | 100.00 | 100.00 |
| *Pearson* χ*^2^ = 0.647, p= 0.421; Cramer's V= 0.109.* | | | | | |

| **Table S6. Distribution of different combinations of incoherence among laypeople.** | | | | | |
| --- | --- | --- | --- | --- | --- |
|  | | | *Internal coherence* | | |
|  |  |  | *0* | *1* | *Total* |
| *External coherence* | *0* | Frequency | 23 | 12 | 35 |
|  |  | Row percentage | 65.71 | 34.29 | 100.00 |
|  |  | Column percentage | 14.56 | 8.28 | 11.55 |
|  | *1* | Frequency | 135 | 133 | 268 |
|  |  | Row percentage | 50.37 | 49.63 | 100.00 |
|  |  | Column percentage | 85.44 | 91.72 | 88.45 |
|  | *Total* | Frequency | 158 | 145 | 303 |
|  |  | Row percentage | 52.15 | 42.59 | 100.00 |
|  |  | Column percentage | 100.00 | 100.00 | 100.00 |
| *Pearson* χ*^2^ = 2.920, p= 0.088; Cramer's V= 0.098.* | | | | | |
