# Supplementary figures and images for: Effect of Class II functional treatment on facial attractiveness, as perceived by professionals and laypeople
Source: Sci Rep. 2021 Jul 7;11:13989. doi: 10.1038/s41598-021-93343-0 (PMC8263773; doi:10.1038/s41598-021-93343-0)

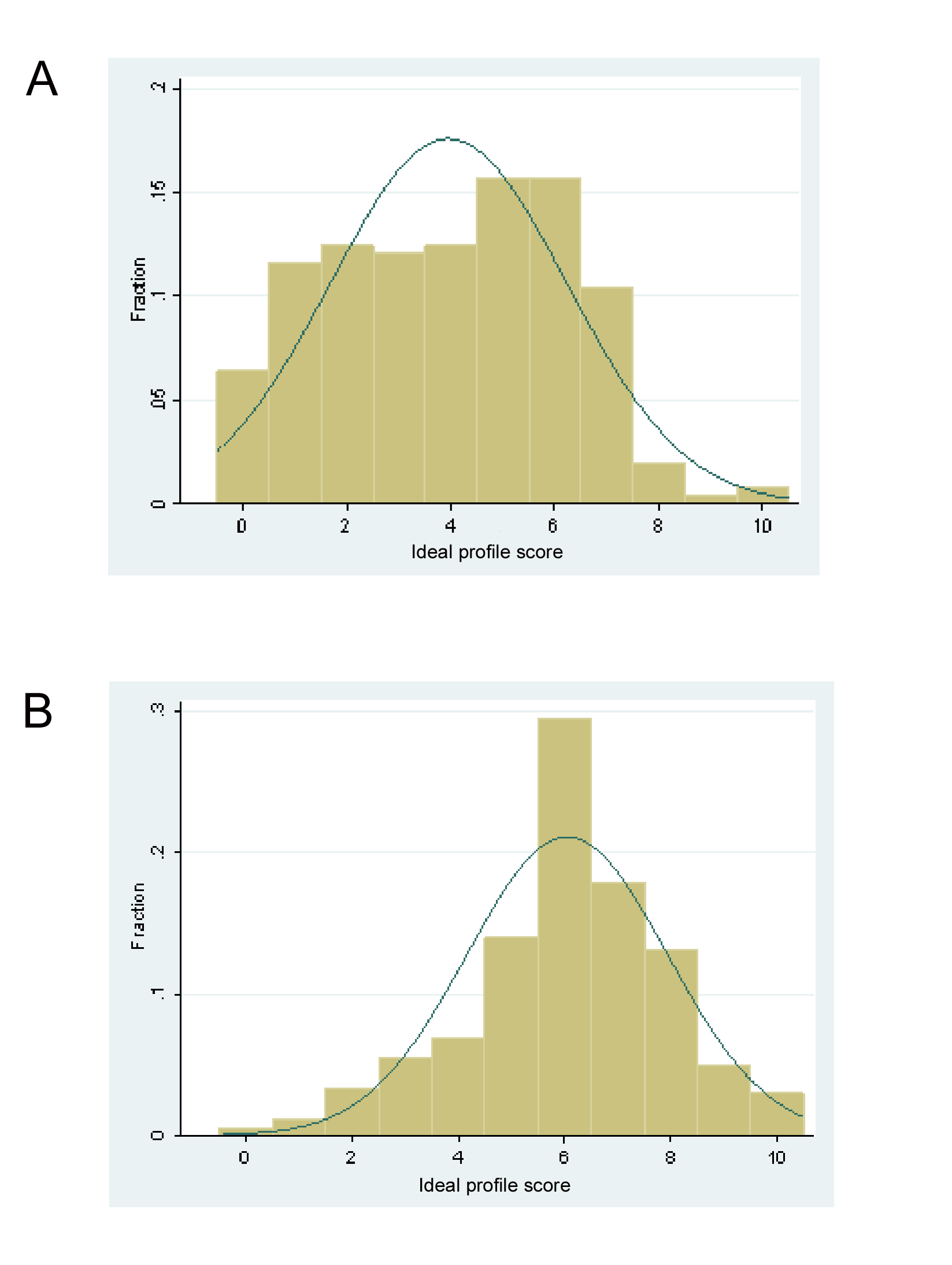

Supplement: Supplementary file 3 — Supplementary Information 2. [file 41598_2021_93343_MOESM3_ESM.tif]
